# The inter-individual anatomical variation of the trochlear notch as a predisposition for simple elbow dislocation

**DOI:** 10.1007/s00402-021-04284-2

**Published:** 2021-12-25

**Authors:** Sofia Kilgus, Christian Eder, Paul Siegert, Philipp Moroder, Elke Zimmermann, Kathi Thiele

**Affiliations:** 1 Center for Musculoskeletal Surgery, Charité-University Medicine Berlin, Augustenburger Platz 1, Berlin, 13353 Germany; 2 Orthopedic Hospital Speising, Speisinger Street 109, 1130 Vienna, Austria; 3 Department of Radiology, Charité-University Medicine Berlin, Chariteplatz 1, Berlin, 10117 Germany

**Keywords:** Elbow dislocation, Trochlear notch, Anatomical variance, Proximal ulna, Elbow instability, Predisposition

## Abstract

**Purpose:**

Besides the multi-layered capsule–ligamentous complex of the elbow joint the high bony congruence in the ulnohumeral joint contributes to elbow stability. Therefore, we assume that specific anatomical configurations of the trochlear notch predispose to dislocation. In case of ligamentous elbow dislocation both conservative and surgical treatment is possible without a clear treatment algorithm. Findings of constitutional bony configurations could help deciding for the best treatment option.

**Methods:**

In this retrospective matched-pair analysis we compared MRI imaging from patients sustaining a primary traumatic elbow dislocation (instability group) with patients suffering from chronic lateral epicondylitis (control group), treated between 2009 and 2019. Two independent observers measured different anatomical landmarks of the trochlear notch in a multiplanar reconstructed standardized sagittal trochlear plane (SSTP). Primarily, opening angle and relative depth of the trochlear notch were determined. After adjustment to the proximal ulnar rim in the SSTP, coronoid and olecranon angle, the articular angle as well as the ratio of the tip heights of the trochlear notch were measured.

**Results:**

We compared 34 patients in the instability group (age 48 ± 14 years, f/m 19/15) with 34 patients in the control group (age 47 ± 16 years, f/m 19/15). Instability group showed a significantly larger opening angle (94.1° ± 6.9° vs. 88.5° ± 6.9°, *p *= 0.0002), olecranon angle (60.9° ± 5.3° vs. 56.1° ± 5.1°, *p *< 0.0001) and articular angle (24.7° ± 6.4° vs. 22.3° ± 5.8°, *p *= 0.02) compared to the control group. Measuring the height from the coronoid (ch) and olecranon (oh) tip also revealed a significantly larger tip ratio (tr = ch/oh) in the instability group (2.7 ± 0.8 vs. 2.2 ± 0.5, *p *< 0.0001). The relative depth (61.0% ± 8.3% vs. 62.7% ± 6.0%, *p *= 0.21) of the trochlear notch as well as the coronoid angle (32.8° ± 4.5° vs. 31.7° ± 5.2°, *p *= 0.30) showed no significant difference in the instability group compared to the control group. The interrater reliability of all measurements was between 0.83 and 0.94.

**Conclusion:**

MRI of patients with elbow dislocation show that there seems to be a bony anatomical predisposition. According to the results, it seems reasonable to include predisposing bony factors in the decision-making process when surgical stabilization and conservative treatment is possible. Further biomechanical studies should prove these assumptions to generate critical bony values helping surgeons with decision making.

**Level of evidence:**

III.

## Introduction

Acute “simple” elbow dislocations (without accompanying bony injury) occur primarily in a physically active group of patients. Although non-surgical therapy is propagated as standard, the significance of a primarily surgical procedure is currently unclear. In a systematic review, published by Hackl et al. in 2015, the results of conservative and operative therapy of simple elbow dislocations were analyzed [8]. Concerning the results of this meta-analysis there are no evidence-based indication criteria for a primary surgical procedure. However, if the good to excellent objective results after conservative therapy are critically compared with the subjective, patient-based results, more than 50% of the patients still report persistent pain and stiffness in the mid-term course [1, 3, 10, 11]. For this reason, alternative surgical techniques such as ligament refixation with or without augmentative ligament bracing are favored in cases of higher degrees of instability or functional demands.

There are still no clearly defined values indicating surgical treatment of ligamentous injury patterns. Different approaches try to adapt the need of surgery to the grade of instability. Fluoroscopic stress images with graduation of varus and valgus instability [18], as well as direct and indirect radiological parameters such as the drop sign, joint incongruity and signs of rotational instability are included in the decision-making process [5]. Previous algorithms aim to quantify the soft tissue damage to make a therapeutic decision and recommend surgery especially for injuries affecting ligamentous and muscular structures together [16].

Besides soft tissue damage, another criterion for or against surgery could be individual constitutional bony configuration, which might predispose to instability. The elbow joint is characterized by a high bony congruency, especially in the ulnohumeral articulation [12]. Previous clinical and biomechanical studies focus on coronoid fracture treatment and identified the stabilizing osseous parts of the humeroulnar joint by stepwise excision of the proximal ulna. Based on the previous findings, the bony configuration of the trochlear notch appears to be a decisive factor in determining elbow stability [2, 14]. The opening angle between the coronoid process and the olecranon tip in particular is the focus of interest in this study. Evidence for an anatomical variation of the angulation of the trochlear notch was already described in 1986 by Wadström et al. [19]. In their study they demonstrated a possible correlation between a bony configuration of the proximal ulna and the dislocation mechanism of the elbow joint based on X-rays.

The aim of this work is to uncover constitutional differences in bony configuration of the trochlear notch, which can promote elbow instability. We, therefore, measured different parameters on a defined MRI imaging plane and compared unstable to stable elbow joints. Our hypothesis was that patients with elbow dislocation have a constitutionally altered angulation and smaller concavity of the trochlear notch than controls.

## Methods

### Study design and patients

This retrospective matched-pair single institution analysis investigated individual differences in the bony configuration of the trochlear notch in two elbow joint pathologies. An MRI-based evaluation of patients with elbow dislocation was conducted (instability group). MRI examinations of patients with chronic lateral epicondylitis served as a comparison group (control group). Data were collected from 2009 to 2019 in a tertiary referral center for elbow pathologies. The study protocol was approved by the local ethics committee (registration number: EA2/235/17). The inclusion criteria for the instability group were (1) a minimum age > 18 years, (2) sustained acute traumatic elbow dislocation and 3) available MRI imaging. Inclusion criteria for control group were (1) a minimum age > 18 years, (2) suffering from chronic lateral epicondylitis and (3) available MRI imaging. Patients with concomitant bony injuries, post-traumatic deformities or cubital osteoarthritis were excluded. In addition, in the control group, all patients with hints of elbow instability in medical history, clinical examination or MRI were excluded. Forty-one (instability group) and 53 patients (control group) were included after retrospective identification regarding study participation suitability based on available data.

### Study parameters and three-dimensional (3D) magnetic resonance imaging (MRI) reconstruction

MRI was performed on a high-field scanner (1.5-T Expree; Siemens Erlangen, Germany) for the right or left elbow using a dedicated knee coil (8 channels), with the arm positioned in full extension and, therefore, in complete supination for the group of epicondylitis. The positioning of the elbow in the dislocation group varied according to the symptoms and the degree of instability, so that images are also available in a flexed position. This could possibly interfere with the standardization of the measurements. Three-dimensional balanced steady-state free precession images were acquired on the axial, sagittal, and coronal planes. The measurements were based on T2-weighted sagittal sequences. All data sets were transferred to a dedicated workstation (Visage 7 software, Visage Imaging GmbH, Berlin, Germany, 2018) for post-processing and analysis. Two observers independently performed all the measurements.

**Fig. 1:**
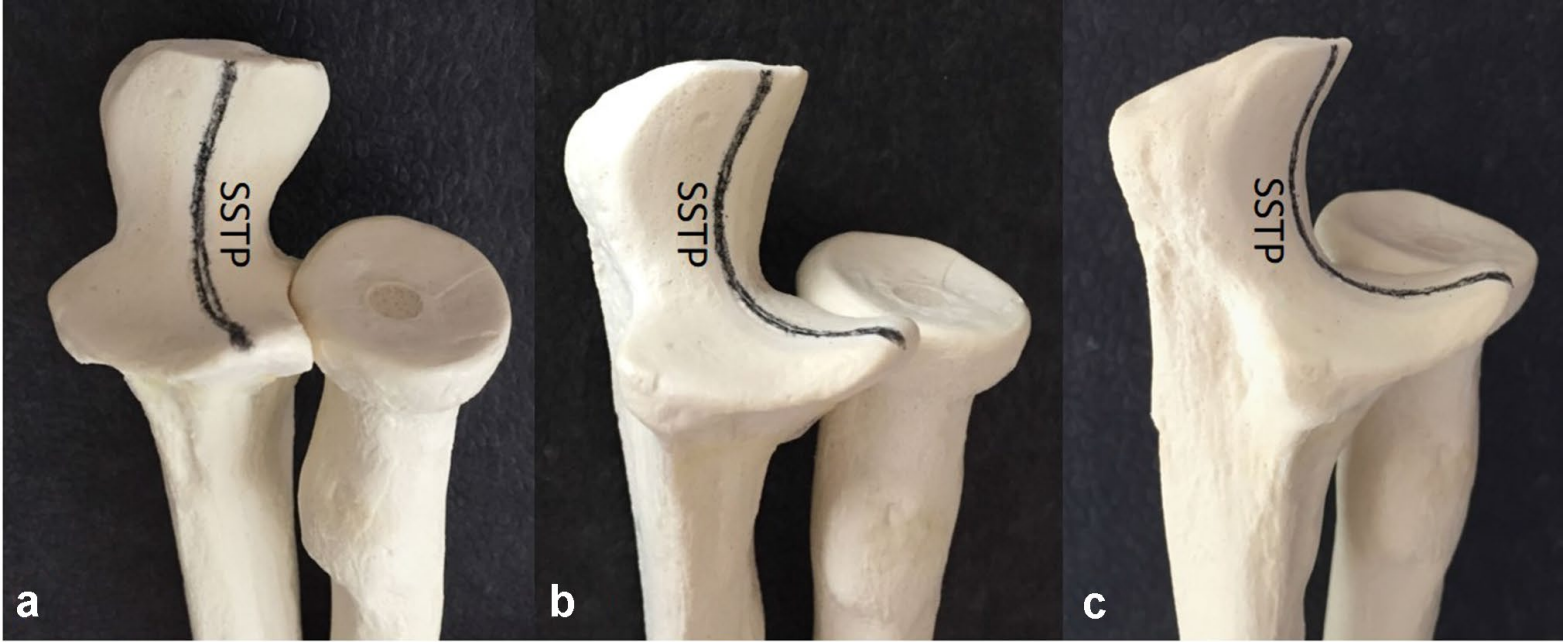
Standardized sagittal trochlear plane (SSTP) for measurement. The plane, used for measurement, is between the coronoid and olecranon tip and shows the smallest radius, demonstrated in the anteroposterior (**a**), oblique (**b**) and lateral view (**c**)

As the trochlear notch has a variable depth in the sagittal plane, we used a multiplanar three-dimensional reconstruction to find a standardized sagittal trochlear plane (SSTP) between the coronoid process and the olecranon tip, which has got the smallest articular radius (Fig. 1).

**Fig. 2:**
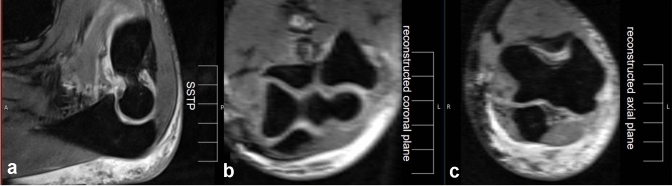
Three-dimensional reconstruction in magnetic resonance imaging (MRI). To find the standardized sagittal trochlear plane (SSTP) between the tip of the olecranon and coronoid (**a**), a multiplanar reconstruction with a coronal (**b**) and an axial plane (**c**) was performed

To find the corresponding plane, a multiplanar 3D reconstruction was performed using the axial and coronal plane (Fig. 2).

**Fig. 3:**
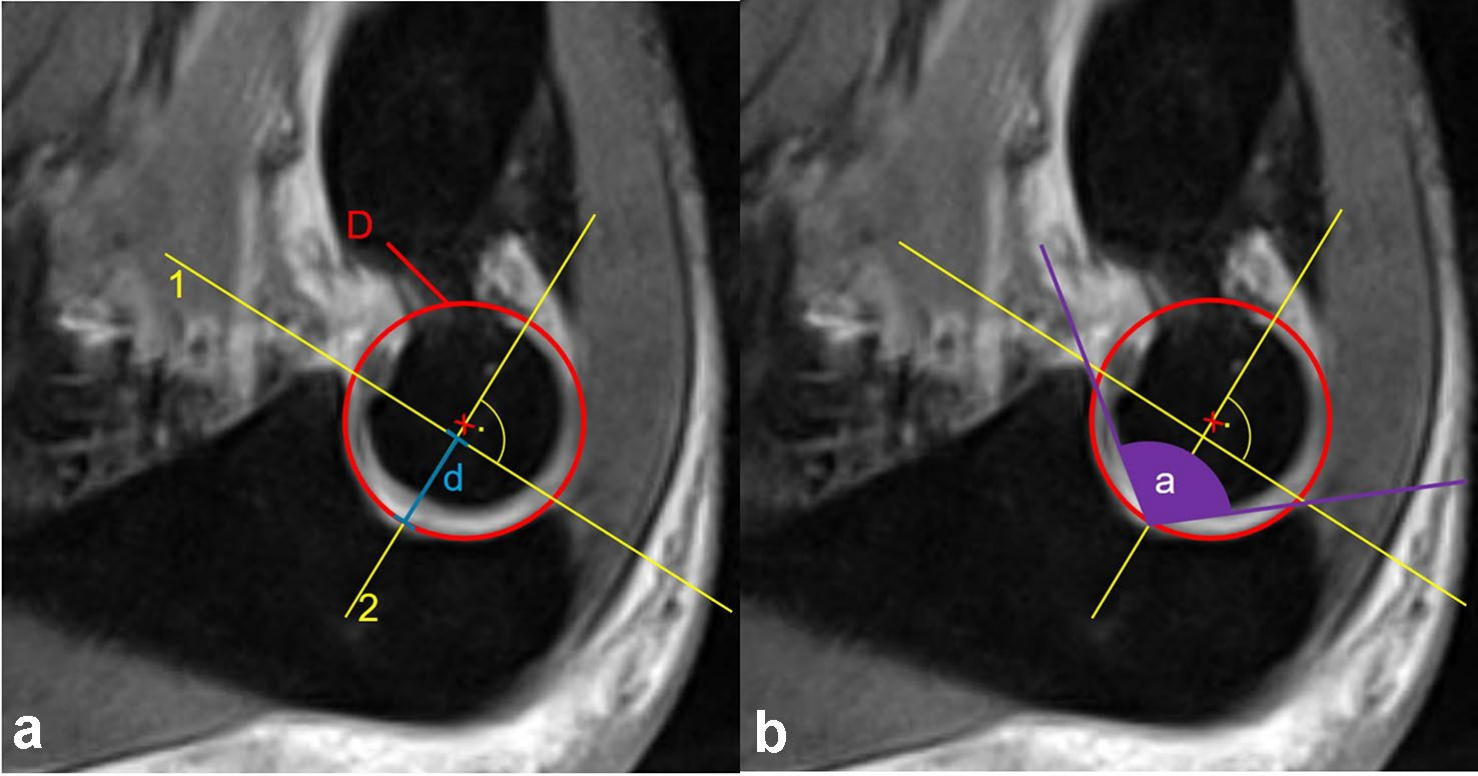
Drawings for calculation of the relative depth and opening angle in the standardized sagittal trochlear plane (SSTP). A best fit circle (red) is drawn in the trochlear notch. Line 1 connects the olecranon and coronoid tip. Line 2 is drawn perpendicularly. Diameter (D) of the best fit circle is calculated automatically by Visage software. **a** Depth of the trochlear notch (**d**) is drawn. **b** The opening angle (**a**) is determined by the help of a line 1, connecting the olecranon and coronoid tip and the distinction point of line 2 with best fit circle

After determining this standardized sagittal trochlear plane (SSTP), measurements were performed (Fig. 3). First a best fit circle was applied on the articular surface of the trochlear notch and the diameter (D) was calculated by the Visage Software. Then a line 1 between the bony contours of coronoid process and olecranon tip was drawn. Ninety degrees to line 1, line 2 was drawn, which crosses the midpoint of the best fit circle. The depth (d) of the trochlear notch is determined on line 2. It is the distance between line 1 and the best fit circle. The opening angle (a) of the trochlear notch is drawn between the two tips and the point, where line 2 crosses the best fit circle.

**Fig. 4:**
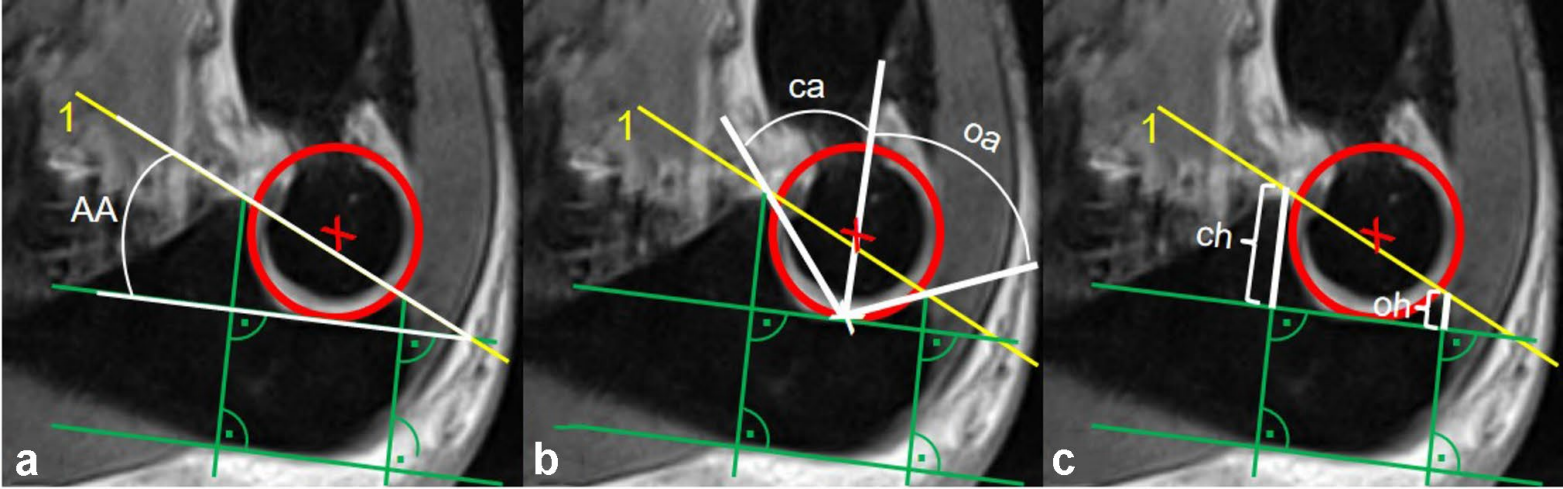
Drawings for calculation of the articular angle (AA), adjusted opening angles and tip heights in the standardized sagittal trochlear plane (SSTP). A line (green) is drawn along the dorsal rim of the proximal ulna, which marks an adjusted intersection point (white cross) on the best fit circle. **a** AA is calculated between the green line and line 1. **b** Adjusted opening angles are drawn from the previously determined intersection point. The coronoid angle (ca) intersects the coronoid tip and the best fit circle midpoint, the olecranon angle (oa) intersects the olecranon tip and the best fit circle midpoint. **c** Coronoid height (ch) and olecranon height (oh) are measured

To consider the orientation of the trochlear notch to the physiological dorsal (PUDA) and varus angulation (VA) of the proximal ulna, a line (green) is drawn along the dorsal rim of the proximal ulna (Fig. 4). By the help of a parallel line, an adjusted intersection point (white cross) on the best fit circle can be marked. Those patients with MRI, in which safe and exact orientation along the dorsal rim was not possible, were excluded from the following measurements. After drawing the two parallel green lines, the articular angle (AA) [4] was calculated between the green line, intersecting the best fit circle, and line 1 (Fig. 4a). Then the adjusted opening angles were drawn from the previously determined intersection point. The coronoid angle (ca) intersects the coronoid tip and the best fit circle midpoint, the olecranon angle (oa) intersects the olecranon tip and the best fit circle midpoint (Fig. 4b). For better understanding and comparability, the terms (ca) and (oa) were chosen. However, it must be pointed out, that the geometric dimensioning does not correspond to anatomical nomenclature (intersection point does not correspond to the bare area). In addition, distance from the green parallel line to the tips were measured and defined as coronoid height (ch) and olecranon height (oh) (Fig. 4c).

### Calculation and statistics

Relative depth (r) was computed by dividing depth (d) through the diameter of the best fit circle (D) (r = d/D) and was specified as a percentage of the total diameter. The angle measurements of the opening angle (a), articular angle (AA), coronoid (ca) and olecranon (oa) angle were drawn with Visage 7 software and have been calculated automatically. Coronoid and olecranon height were drawn and measured by Visage Software with the same digitally scaled unit. Then tip ratio (tr) was calculated by dividing coronoid height (ch) through olecranon height (oh) (tr = ch/oh).

Two raters conducted the measurements independently at different time points. Intra-class correlation coefficient (ICC) with a 95% confidence interval (CI) was calculated for all measurements. As recommended by Landis and Koch, an ICC < 0.20 resembles slight, 0.21 to 0.40 fair, 0.41 to 0.60 moderate, 0.61 to 0.80 substantial, and > 0.81 almost perfect agreement. After reliability assessment, values of both raters were averaged for further analysis.

A matched-pair analysis was performed. Patients of the two groups were matched on gender (f/m) and age (± 12 years). The Wilcoxon signed-rank test was used in case of nonparametric distribution (ca), the paired *t*—test was performed in case of normal distribution (a, oa, AA, tr, r). The results were given as mean and standard deviation (SD) or as numbers and percentage. Statistical analysis was performed using GraphPad Prism Version 8.0 (GraphPad Software, San Diego, CA, United States) and SPSS (IBM SPASS Statistics 25, New York, United States). A *p* value < 0.05 was considered significant.

## Results

### Demographic data

According to the inclusion criteria and matching on gender and age, 34 patients with simple elbow dislocation and 34 patients with lateral epicondylitis were measured. The demographic data can be found in Table 1.

**Table 1:** General characteristics of the instability (elbow dislocation) and the control group (lateral epicondylitis), yr = year, f = female, m = male, r = right, l = left

	Instability group	Control group	*p* value
	*n* = 34	*n* = 34	
Age (yr)	48 ± 14	47 ± 16	0.72
Sex (f/m)	19/15	19/15	
Side (r/l)	17/17	21/13	

### Measurement

The following measurements were taken in sagittal sequences of all patients (Table 2). All measurements show almost perfect agreement between the two raters. Intra-class correlation coefficients (ICC) are summarized in Table 2.

**Table 2:** Results of the opening angle (a), relative depth (r), articular angle (AA), coronoid angle (ca), olecranon angle (oa), tip ratio (tr) include *n* = 34 (instability group) and *n* = 34 (control group)

	Rater	Mean	SD	95% Confidential Interval		ICC	Agreement
				Lower bound	Upper bound		
Opening angle (a)						0.88	Almost perfect
Instability group	R1	94.7	7.2	92.2	97.2		
Instability group	R2	93.4	6.6	91.1	95.8		
Control group	R1	88.3	6.7	86.0	90.6		
Control group	R2	88.8	7.2	86.3	91.3		
Relative depth (r)						0.83	Almost perfect
Instability group	R1	60.4	8.0	57.6	63.1		
Instability group	R2	61.6	8.7	58.6	64.7		
Control group	R1	63.3	6.4	61.0	65.5		
Control group	R2	62.2	5.6	60.2	64.1		
Articular angle (AA)						0.94	Almost perfect
Instability group	R1	24.3	6.7	22.0	26.6		
Instability group	R2	25.0	6.1	22.9	27.2		
Control group	R1	22.2	5.9	20.1	24.2		
Control group	R2	22.5	5.7	20.5	24.4		
Coronoid angle (ca)						0.87	Almost perfect
Instability group	R1	32.7	4.5	31.1	34.2		
Instability group	R2	32.9	4.6	31.3	34.5		
Control group	R1	31.5	5.0	29.8	33.2		
Control group	R2	32.0	5.4	30.1	33.9		
Olecranon angle (oa)						0.89	Almost perfect
Instability group	R1	60.3	5.5	58.4	62.2		
Instability group	R2	61.4	5.1	59.6	63.2		
Control group	R1	55.3	5.7	53.4	57.3		
Control group	R2	56.8	4.4	55.2	58.3		
Tip ratio (tr)						0.94	Almost perfect
Instability group	R1	2.7	0.8	2.4	2.9		
Instability group	R2	2.8	0.8	2.5	3.1		
Control group	R1	2.1	0.5	2.0	2.3		
Control group	R2	2.3	0.5	2.1	2.4		

Values of the two raters (R1, R2) measured in the standardized sagittal trochlear plane [mean, standard deviation (SD), 95% confidential interval, intra-class correlation coefficient (ICC), agreement]

**Fig. 5:**
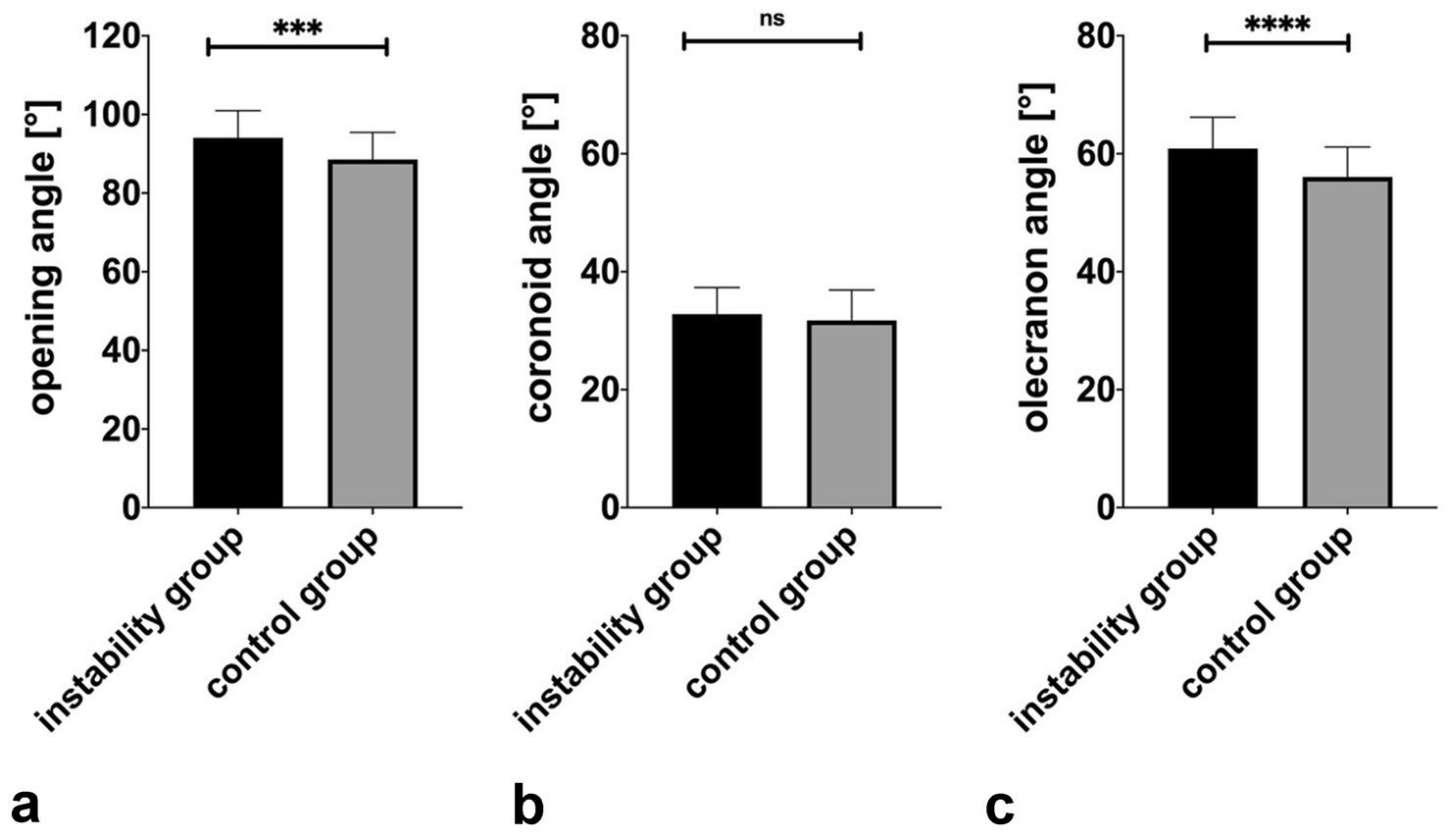
Display of the MRI results regarding the opening angle and the partial angles (coronoid and olecranon angle) of the trochlear notch in the standardized sagittal trochlear plane (SSTP). Values of both raters are averaged. **a** Opening angle: a larger opening (*p *= 0.0002) is measured in patients with elbow dislocation. **b** Coronoid angle: no significant difference of the coronoid angle was found (*p *= 0.30) **c** Olecranon angle: a significantly wider olecranon angle (*p *< 0.0001) is measured in patients with elbow dislocation. ***p < 0.001, ****p < 0.0001, ns non-significant

After reliability assessment, values of both raters were averaged for further analysis. A direct comparison of both pathologies showed a significantly larger opening angle of 94.1° (range 81.7–116.4°, SD 6.9°) in the group of elbow dislocation compared to the group of lateral epicondylitis with 88.5° (range 68.1–105.0°, SD 6.9°), *p *= 0.0002. Considering the partial angles in detail, according to the definition of an independent vertex (intersection point), the adjusted olecranon angle is significantly wider in the instability group with 60.9° (range 51.3–71.6°, SD 5.3°) than in the control group with 56.1° (range 39.0–66.9°, SD 5.1°), *p *< 0.0001. The adjusted coronoid angle revealed no significant difference between the instability group with 32.8° (range 21.4–48.5°, SD 4.5°) and the control group with 31.7° (range 18.8–42.4°, SD 5.2°), *p *= 0.31. These results are shown graphically in Fig. 5.

**Fig. 6:**
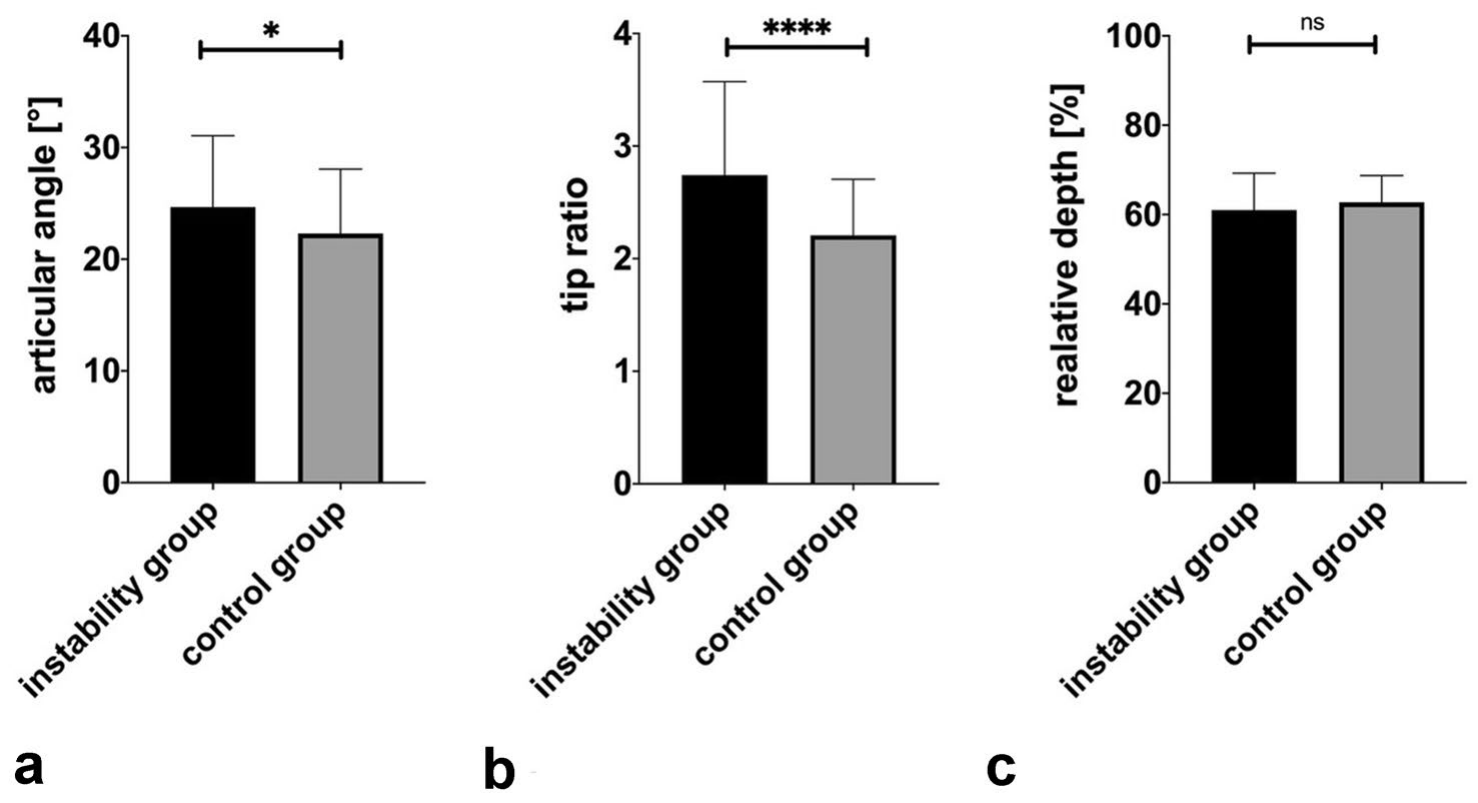
Display of the MRI results in the standardized sagittal trochlear plane (SSTP) regarding the articular angle, tip ratio and relative depth. Values of both raters are averaged. **a** Articular angle: A significantly wider articular angle in the instability is measured than in the control group (*p* = 0.02). **b** Tip ratio: a significantly higher tip ratio is determined in the instability group (*p* < 0.0001). **c** Relative depth: NO significantly lower relative depth in the instability group was seen (*p* = 0.21). *p < 0.05, ****p < 0.0001, ns non-significant

The articular angle was significantly wider in the instability group (mean 24.7°, range 8.7–40.0°, SD 6.4°) than in the control group (mean 22.3°, range 6.3–34.1°, SD 5.8°), *p *= 0.02. Measuring the distance from the coronoid and olecranon tip showed a significantly larger tip ratio (tr) in the instability group with 2.7 (range 0.8–1.4, SD 5.0) compared to the control group with 2.2 (range 1.1–3.5, SD 0.5), *p *< 0.0001. The relative depth of the trochlear notch is not significantly lower with 61.0% (range 36.6–77.9%, SD 8.3%) compared to the control group with 62.7% (range 50.5–79.4%, SD 6.0%), *p *= 0.21. These results are shown graphically in Fig. 6.

## Discussion

**Fig. 7:**
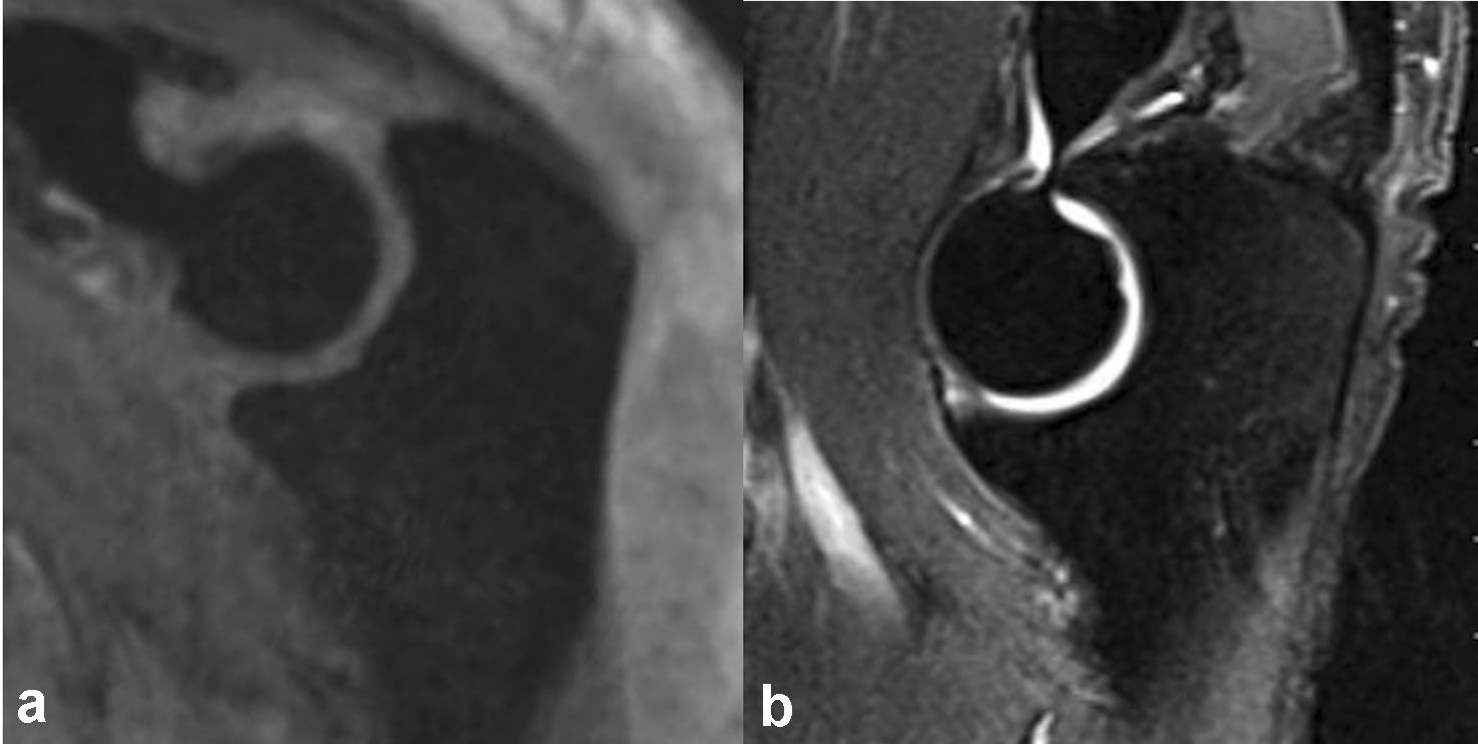
Imaging of two maximum variants of the anatomical alignment of the trochlear notch in the standardized sagittal trochlear plane (SSTP). **a** Clearly large opening angle (103.7°) in a patient with simple elbow dislocation. **b** High ulnohumeral congruency with a small opening angle (83.0°) in a patient with chronic lateral epicondylitis

The treatment of simple elbow dislocation continues to be the subject of controversy with the question of which possible indicators plead for surgical stabilization. The current therapeutic approach considers the extent of the soft tissue damage and the risk of re-dislocation in an elbow position close to extension [20]. The present study pursues the hypothesis that the bony configuration of the trochlear notch has a significant influence on elbow stability. To support this hypothesis, an MRI-based study was performed in patients with simple elbow dislocation compared to patients with a stable elbow joint (chronic lateral epicondylitis). Special attention was paid to different angles and the depth of the notch as well as the coronoid process and olecranon tip. In this study inter-individual variation of the trochlear notch was seen. A significantly wider olecranon and total opening angle as well as a larger articular angle were found in the instability group and leads to the presumption, that a bony predisposition of the trochlear notch facilitates a dislocation of the elbow joint. Therefore, we also assume that two maximum bony variants can be differentiated based on their respective shape of curvature: either a wide (Fig. 7a) or a narrow type (Fig. 7b).

The findings of this study about the anatomy of the trochlear notch could be used as additional criterion for the assessment of elbow stability, as the ulnohumeral joint is one of the primary constraints besides the MCL, LCL and radial head [12, 15, 17]. Morrey et al. showed that the articular and ligamentous contribution to the stability of the elbow differ during motion while joint articulation can offer up to 75% of stability [13].

For reproducible analysis and, therefore, estimating the stability of the ulnohumeral joint, we introduce a 3D reconstructed sagittal plane (SSTP). This created plane between the coronoid and olecranon tip also respects the physiological varus and dorsal bow of the proximal ulna.

Other morphological studies also dealt with the anatomical variation of the trochlear notch, however, without specific focus on elbow instability. By the help of three-dimensional digitizing system and coordinates of the landmarks in 35 cadaveric arms, Matzon et al. analyzed the relative coronoid height (41–48% of the ulnar height) and the angulation between coronoid and olecranon (“open angle”) in relation to the distal coronoid border (33°, SD 3°) and the insertion of the brachial muscle (38°, SD 4°) [9]. Matzon’s study exclusively deals with the question of gender-specific differences (no differences), but already discusses a possible correlation with recurrent instability at lower open angles as well as the good reproduction of the measurement method in conventional radiographs [9].

Giannicola et al. also analyzed 78 healthy elbows in two MRI-based studies with the question of bony and chondral distribution in the trochlear notch. In doing so, they crystallized two anatomical phenotypes in both the coronal and the sagittal plane. In the coronal plane, the trochlear notch angle (TNA) is defined as the angle formed by the depth of the sulcus and two ridges and measured between 124° and 156°. Therefore, the authors assume the existence of a curved-shaped trochlea (TNA < 142°) and a flat-shaped trochlea (TNA > 142°) without making further comments on the stability of the joint [6]. In the sagittal view, two morphological types were also described with the definition of the "Greater Sigmoid Notch Coverage Angle" (GSN-ca). GSN-ca is the sum of bony and cartilaginous angles of the trochlear notch, whose center is the humeral trochlea [7]. The authors identified a coverage angle of more than 180° (“closed type”) in 92% of their cases, whereas in 8% of the cases the rare "open type" GSN-ca was found [7]. Following basic geometric principles, the geometrics constraint limits the relative displacement between the 2 components of the ulnohumeral articulation and allows rotation only. Accordingly, a smaller GSN-ca is assumed to provide a greater range of motion, which could predispose to elbow instability. However, a direct correlation could not be further proven in this study due to the fact that exclusively a healthy population was considered.

In continuation of the previous study results, our work focuses on the direct comparison of the bony configuration of the trochlear notch in patients with an acute elbow dislocation and a control group without a dislocation event or known preexisting instability. Our data confirm the assumption of Giannicola et al., who discussed a predisposition for elbow joint instability by describing the “open type” [7].

There are only few studies about simple elbow dislocations and the anatomical shape of the proximal ulna. A direct correlation between the anatomical bony variation of the trochlear notch and instability in elbow joints was shown by Wadström et al. with the evaluation of 100 dislocated elbow joints compared to 150 elbow joints without previous injury in a strict lateral view in 2D radiography [19]. The radiological evaluation showed that the angle (a), formed by the ulnar shaft axis and the connecting line of the coronoid process and the tip of the olecranon, was significantly smaller compared to the control group (median 24° vs. 25°). The central angle (b) of the semilunar notch (= trochlear notch), formed by the midpoint of the best fit circle and the coronoid process and olecranon tip, was significantly larger in case of an elbow dislocation (median 184° vs. 178°). The authors concluded on a possible existence of two forms of trochlear notch with a small “a”—angle, a large central angle and a low coronoid height, mainly in the group of elbow dislocation. The authors explain the surprisingly larger central angle (b) in the dislocation group with an olecranon tip extended far ventrally and discuss it as an increased hypomochlion in hyperextension during the dislocation.

In contrast to these findings, our study results show the formally hypothesized bony predisposition with a significantly larger opening angle, which Giannicola et al. had already postulated in their study [7]. We believe that the “open type” not only allows a general higher range of motion but also a proportionally greater hyperextension. Especially after analyzing the partial angles with a significantly larger angle fraction towards the olecranon, this thesis can be supported. Because of the important stabilizing role of the olecranon in extension and in combination with a typical trauma mechanism for elbow dislocation [16], a wide olecranon angle possibly facilitates hyperextension and, therefore, dislocation. The significantly higher tip ratio in the instability group implies a relatively larger sagittal extension of the coronoid process. We assume a compensatory bony support to counteract the dislocation tendency in case of other anatomical predispositions, especially if the olecranon angle is enlarged.

This contradicts the dimensioning of the central angle and the measurements of the coronoid heights according to Wadström et al. as well as their explanation. Yet, in contrast to Wadström et al., in our study, measurements were performed in the 3D reconstructed standardized sagittal trochlear plane (SSTP) and with additional adjustment to the dorsal and varus angulation of the proximal ulna. Wadström et al. orientated their measurements to the longitudinal axis of the ulna in a single 2D fluoroscopy image. Furthermore, it does not respect the physiological variation of the notch’s depth in the sagittal plane from lateral to medial. The necessity of a strictly lateral X-ray image of the acutely traumatized elbow is also a complicating factor for implementation in everyday clinical practice. Overall, the numerical variances described by Wadström et al. between the control and pathology group are proportionally very small (“a”—angle 1° difference in median, “coronoid height” identical median). In addition, the olecranon height in general and its correlation to the coronoid has not been measured.

With regard to the limitations of our studies, some of the results must be interpreted with caution when numerical differences are only small concerning the anatomical variation of the trochlear notch. Possible reason is, that the control group can also include patients with bony instability predisposition who have not experienced appropriate trauma for dislocation before. In addition, it has to be considered that the control group is rather small.

An MRI-based evaluation was carried out, although bony structures were defined. This is mainly due to the clinical pathologies which have to be examined, since ligamentous injury patterns can be better quantified in MRI than in a CT scan. Nevertheless, todays’ high-field scanners also enable MRI-based differentiation between chondral and bony layers. Our data present the bony configuration only, as we assume the bone as a real static constraint. To which extent the cartilaginous contribution is particularly relevant in cases with an “open”, respectively, a “wide” type cannot be clarified in this study.

To increase the statistical power of our results, the study is based on the design of a matched paired analysis. However, the age variance must be mentioned as a limitation, since the two considered pathologies are typically found in different age groups.

## Conclusion

In conclusion, our data show that there seems to be a bony anatomical predisposition for dislocation in the proximal ulna. We suspect that the lower bony coverage can cause instability in the elbow joint. In our opinion the significantly larger opening angle facilitate a dislocation. According to the results, it seems reasonable to include predisposing bony factors in the decision-making process for surgical stabilization in addition to the quantification of soft tissue damage. Further biomechanical studies should prove this assumption to generate critical bony values which can help surgeons with decision-making. It must be noted that the study cannot make any statements about the extent to which a bony defect situation can be offset by a ligamentous reconstruction.

## References

[1] Anakwe RE, Middleton SD, Jenkins PJ, McQueen MM, Court-Brown CM (2011). Patient-reported outcomes after simple dislocation of the elbow. J Bone Joint Surg Am.

[2] Beingessner DM, Dunning CE, Stacpoole RA, Johnson JA, King GJW (2007). The effect of coronoid fractures on elbow kinematics and stability. Clin Biomechanics.

[3] Beirer M, Friese H, Lenich A, Crönlein M, Sandmann GH, Biberthaler P (2017). The Elbow Self-Assessment Score (ESAS): development and validation of a new patient-reported outcome measurement tool for elbow disorders. Knee Surg Sports Traumatol Arthrosc.

[4] Beşer CG, Demiryürek D, Özsoy H, Erçakmak B, Hayran M, Kızılay O (2014). Redefining the proximal ulna anatomy. Surg Radiol Anat.

[5] Coonrad RW, Roush TF, Major NM, Basamania CJ (2005). The drop sign, a radiographic warning sign of elbow instability. J Shoulder Elbow Surg.

[6] Giannicola G, Scacchi M, Sedati P, Gumina S (2016). Anatomical variations of the trochlear notch angle: MRI analysis of 78 elbows. Musculoskelet Surg.

[7] Giannicola G, Sedati P, Cinotti G, Bullitta G, Polimanti D (2015). The ulnar greater sigmoid notch “coverage angle”: bone and cartilage contribution. Magnetic resonance imaging anatomic study on 78 elbows. J Shoulder Elbow Surg.

[8] Hackl M, Beyer F, Wegmann K, Leschinger T, Burkhart KJ, Müller LP (2015). The treatment of simple elbow dislocation in adults. Dtsch Ärzteblatt.

[9] Matzon JL, Widmer BJ, Draganich LF, Mass DP, Phillips CS (2006). Anatomy of the coronoid process. J Hand Surg [Am].

[10] Mayne IP, Wasserstein D, Modi CS, Henry PDG, Mahomed N, Veillette C (2015). The epidemiology of closed reduction for simple elbow dislocations and the incidence of early subsequent open reduction. J Shoulder Elbow Surg.

[11] Modi CS, Wasserstein D, Mayne IP, Henry PDG, Mahomed N, Veillette CJH (2015). The frequency and risk factors for subsequent surgery after a simple elbow dislocation. Injury.

[12] Morrey BF, An K-N (2005). Stability of the elbow: osseous constraints. J Shoulder Elbow Surg.

[13] Morrey BF, An KN (1983). Articular and ligamentous contributions to the stability of the elbow joint. Am J Sports Med.

[14] O'Driscoll SW, Jupiter JB, Cohen MS, Ring D, McKee MD (2003). Difficult elbow fractures: pearls and pitfalls. Instr Course Lect.

[15] O'Driscoll SW, Jupiter JB, King GJ, Hotchkiss RN, Morrey BF (2001). The unstable elbow. Instr Course Lect.

[16] Robinson PM, Griffiths E, Watts AC (2017). Simple elbow dislocation. Shoulder Elbow.

[17] Safran MR, Baillargeon D (2005). Soft-tissue stabilizers of the elbow. J Shoulder Elbow Surg.

[18] Schnetzke M, Aytac S, Studier-Fischer S, Grützner P-A, Guehring T (2015). Initial joint stability affects the outcome after conservative treatment of simple elbow dislocations: a retrospective study. J Orthop Surg and Res.

[19] Wadström J, Kinast C, Pfeiffer K (1986). Anatomical variations of the semilunar notch in elbow dislocations. Arch Orthop Trauma Surg.

[20] Watts AC (2019). Primary ligament repair for acute elbow dislocation. J Bone Joint Surg Essent Surg Tech.

